# Brain magnetic resonance imaging review suggests unrecognised hypoglycaemia in childhood

**DOI:** 10.3389/fendo.2024.1338980

**Published:** 2024-03-28

**Authors:** Chris Worth, Pon Ramya Gokul, Katie Ramsden, Sarah Worthington, Maria Salomon-Estebanez, Amit Maniyar, Indraneel Banerjee

**Affiliations:** ^1^ Department of Paediatric Endocrinology, Royal Manchester Children’s Hospital, Manchester, United Kingdom; ^2^ Department of Radiology, Royal Manchester Children’s Hospital, Manchester, United Kingdom; ^3^ Faculty of Biology, Medicine and Health, University of Manchester, Manchester, United Kingdom

**Keywords:** hypoglycaemia, child, magnetic resonance imaging, brain injury, glucose

## Abstract

**Introduction:**

Neonatal and early-life hypoglycaemia, is a frequent finding but is often non-specific and asymptomatic, making detection and diagnosis challenging. Hypoglycaemia-induced cerebral injury can be identified by magnetic resonance imaging (MRI) changes in cerebral white matter, occipital lobes, and posterior parietotemporal regions. It is unknown if children may have hypoglycaemic brain injury secondary to unrecognised hypoglycaemia in early life. We have examined retrospective radiological findings of likely brain injury by neuroimaging to investigate the existence of previous missed hypoglycaemic events.

**Methods:**

Retrospective MRI data in children in a single tertiary centre, over a ten-year period was reviewed to identify potential cases of unrecognised early-life hypoglycaemia. A detailed search from an electronic radiology repository involved the term “hypoglycaemia’’ from text-based reports. The initial report was used for those who required serial scanning. Images specific to relevant reports were further reviewed by a designated paediatric neuroradiologist to confirm likely hypoglycaemia induced brain injury. Medical records of those children were subsequently reviewed to assess if the hypoglycaemia had been diagnosed prior to imaging.

**Results:**

A total of 107 MR imaging reports were identified for review, and 52 (48.5%) showed typical features strongly suggestive of hypoglycaemic brain injury. Medical note review confirmed no documented clinical information of hypoglycaemia prior to imaging in 22 (42%) patients, raising the likelihood of missed hypoglycaemic events resulting in brain injury.

**Conclusions:**

We have identified the existence of unrecognised childhood hypoglycaemia through neuroimaging review. This study highlights the need for heightened awareness of early life hypoglycaemia to prevent adverse neurological outcomes later in childhood.

## Introduction

Neonatal hypoglycaemia is a common occurrence but exists along a wide spectrum of severity. There is evidence that untreated, or inadequately treated, early life hypoglycaemia may cause later life neurological injury (1). This is particularly prominent when both glucose and alternative brain fuel sources are simultaneously suppressed, such as in patients with congenital hyperinsulinism (2), irrespective of the permanence of disease (3).

The screening process for neonatal hypoglycaemia is subject to significant variation (4). This variation is partly dependent on the adoption of certain criteria for blood glucose testing, as well as differing hypoglycaemia thresholds for further investigation and treatment (5). As blood glucose testing is not universal, and consensus on what constitutes a “normal blood glucose” is not clear, there is a possibility for missed hypoglycaemia, with or without hyperinsulinism (6). Any missed hypoglycaemia has the potential to cause brain injury and result in neurological manifestations such as seizures and developmental delay (7). Clinicians and families living with Congenital Hyperinsulinism have been concerned that current newborn screening methods are not wholly adequate, resulting in missed neonatal hypoglycaemia presenting later as neurodisability (6). Therefore, it is important to examine this missed evidence through retrospective review, including an assessment of brain scans to detect imprints from prior neuroglycopaenia.

Brain injury secondary to hypoglycaemia preferentially affects the parietal and occipital regions of the brain and can be appreciated on magnetic resonance imaging (MRI) scans (8, 9) with high specificity and positive predictive value. MRI changes in these anatomical areas are strongly suggestive of hypoglycaemic injury and do not suggest alternative aetiologies with a high degree of certainty (9, 10). The existence of radiological evidence of brain impact from previously unrecognised hypoglycaemia has not been evaluated and could provide insight into the possibility of missed and detrimental early-life hypoglycaemia. We have undertaken neuroimaging retrospective review to identify such potential radiological evidence for hypoglycaemia.

### Aim

To evaluate for the existence of any case of missed early-life hypoglycaemia as detected radiologically by magnetic resonance neuroimaging.

## Methods

To detect the possibility of brain changes from prior neuroglycopaenia, we undertook a retrospective review of brain MRI reports over a 10-year period (Dec 2011 to Dec 2021) for a quality improvement project investigating imaging outcomes at a single tertiary referral centre (Royal Manchester Children’s Hospital). As the aim was to detect the existence of any (minimum one) case of missed hypoglycaemia, the study design was retrospective, and a quality improvement method was used. The study received approval from the Children’s Clinical Audit Committee at the Royal Manchester Children’s Hospital (registration 13 Feb 2023). The focus of the study was not to derive a comprehensive list of patients with radiological evidence of early-life hypoglycaemia; instead, retrospective sampling over a 10-year period was adopted as a pragmatic method to interrogate a large database to test the hypothesis that there might be patients with radiological evidence of early-life hypoglycaemia that had not been clinically detected (missed hypoglycaemia).

To this effect, an electronic neuroimaging database in Picture Archiving and Communication System (PACS^®^) at the Royal Manchester Children’s Hospital site was reviewed for the search term “hypoglycaemia” in radiology text reports. The search was undertaken by one operator using manufacturer-specified electronic strategy without the need for additional codes for data extraction or the development of a standard operating procedure or manual. A wider search strategy to include “occipital or parietal lobe changes” was not adopted. This was to limit the generation of false positives requiring timely and costly verification.

The search field included all children (under 16 years at the time of data extraction) referred for an MRI scan, regardless of the reason specified in the requesting information. Text reports were obtained from the initial scan if multiple, serial scans were undertaken, and scans included if the findings indicated hypoglycaemic injury. T2-weighted, coronal fluid-attenuated inversion recovery [FLAIR]-weighted imaging MR sequences were used to identify radiological characteristics of hypoglycaemia in occipital and parietal lobes (10, 11). Neuroimaging findings typical of hypoglycaemia were further reviewed by a paediatric neuro-radiologist who re-analysed images to confirm or refute findings of likely hypoglycaemic brain injury. Patients with brain changes not typical of hypoglycaemia were excluded from investigation. As this study examined text reports retrospectively, text varied in style and syntax, and outcome was binary (hypoglycaemia evidence yes/no), it was not possible to test inter-rater reliability. However, the study team neuro-radiologist was designated to review all scans with positive evidence of hypoglycaemia, ensuring cross checking of scan reporting accuracy to reduce false positives and increase the specificity of findings.

All patients identified by text searching were born in St Mary’s Hospital and Managed Clinical Services and adjoining maternity hospitals in the Manchester region where the regional neonatal network guideline based on the British Association for Perinatal Medicine neonatal hypoglycaemia framework was utilised. Therefore, it was assumed that all children with radiological evidence of hypoglycaemia had been subject to a common newborn hypoglycaemia screening process. However, specific perinatal data including glucose measurements were not obtained for all patients.

The patient names of those with confirmed likely hypoglycaemic injury on MRI were then matched with those in hospital electronic health records and an additional database of patients with congenital hyperinsulinism maintained by the Northern Congenital Hyperinsulinism (NORCHI) service manually by one member of the study team. Patients with prior recorded information of hypoglycaemia and/or congenital hyperinsulinism as diagnosis were filtered out as exclusion criteria. Thus, patients with previously documented hypoglycaemia, or those in whom hypoglycemia was considered a possibility by the referring clinician prior to imaging, were excluded. For children born outside the maternity hospital linked to our centre, maternity and neonatal information was not obtained. Patients with no documented history of hypoglycaemia, and those in whom hypoglycaemia was not suggested or suspected on medical review prior to imaging, were included for review. As per quality improvement methodology, parents or carers were not contacted for further information to confirm absence of hypoglycaemia. For study purposes, only text-based clinician interpretation was used to identify early-life hypoglycaemia. A numerical definition of hypoglycaemia was not used, as this would be less relevant in the context of patients without documented hypoglycaemia in the absence of a widely accepted consensus on specific cutoffs.

For a quality improvement study design with focus on the determination of radiological evidence of hypoglycaemia in patients without documented hypoglycaemia, extensive data capture (such as a wide range of demographic variables) was limited to a need-to-know basis in keeping with principles of good governance. This ensured unnecessary data usage beyond the remit of the study design. This study was not designed to test hypoglycaemia severity with the extent of radiological injury as reported elsewhere (12); therefore, patients with information on hypoglycaemia were excluded for analysis of their reports.

## Results

**Figure 1:**
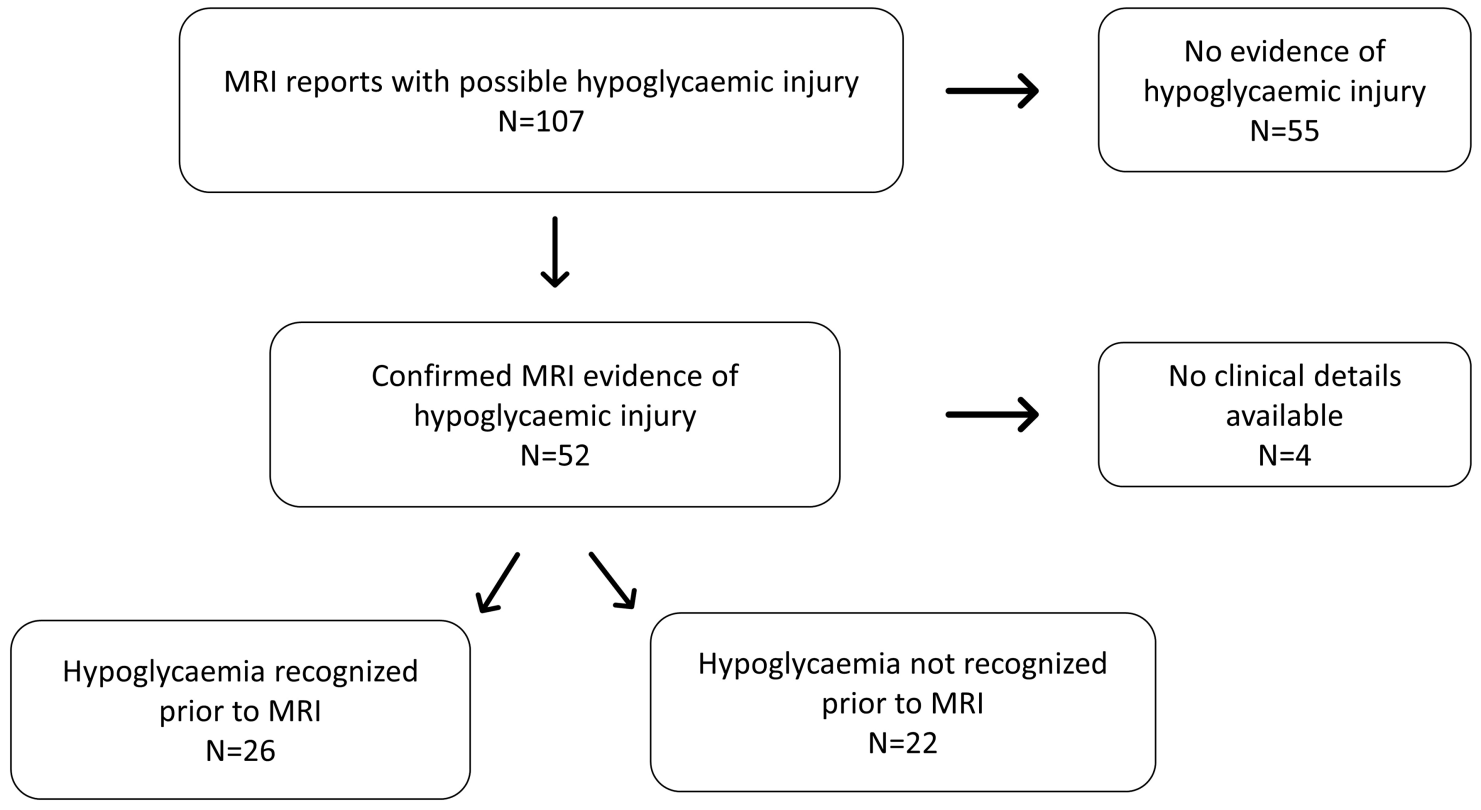
Flowchart diagram illustrating the distribution of patients included in the study (MRI-Magnetic Resonance Imaging).

The initial search for “hypoglycaemia” identified 107 MRI results, of which 52 (48.5%) reported evidence of hypoglycaemic brain injury (**Figure 1**). Seizures, developmental delay and learning difficulties were the most common indications for MR imaging referral. All reported hypoglycaemic brain injury images were confirmed on reanalysis.

At the time of imaging, patients varied in age with a median (range) age of 0.24 (0 –9) years. Review of health records and imaging requests revealed a history of definite or likely hypoglycaemia in 26 (50%) patients. No clinical details were accessible in four patients. In the remaining 22 patients (42% of all scans characteristic of hypoglycaemia) in whom clinical details at referral were available, there was no evidence of: a clinical diagnosis which could include hypoglycaemia; clinical suspicion of hypoglycaemia or; treatment for hypoglycaemia. As individual patients were not contacted, the absence of hypoglycaemia was not confirmed with families. However, these findings strongly suggest that early life hypoglycaemia is likely to have been unrecognised in at least 22 patients over a 10-year period (**Figure 1**).

**Figure 2:**
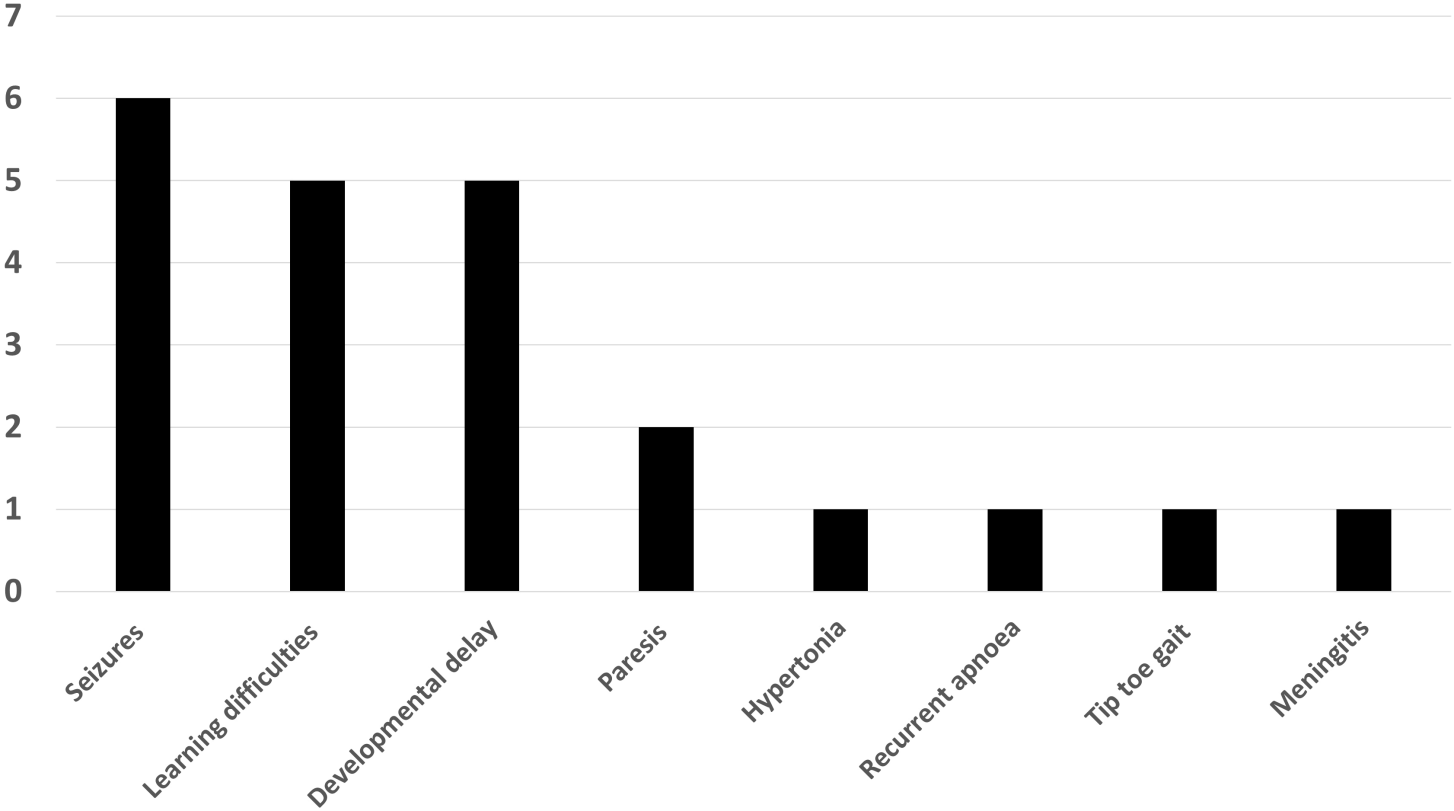
Bar chart showing the distribution of clinical findings in patients with neuroimaging findings suggestive of hypoglycaemic injury.

Patient characteristics, with later life clinical presentation and MRI scan findings have been provided in **Table 1**. Within the missed hypoglycaemia group, 15 (68%) were female and median (range) age was 2.08 (0-14) years. At the time of referral, the following phenotypes were noted – seizures (6), learning difficulties (5), developmental delay (5), paresis (2), hypertonia (1), recurrent apnoea (1), tip toe gait (1) and meningitis (1) (**Figure 2**). All patients had uncomplicated births with no evidence of perinatal stress to suggest possible transient hyperinsulinism. Two patients were born prematurely between 30 and 35 weeks of gestation but no hypoglycaemia was identified during the neonatal period. All patients with imaging findings of hypoglycaemic brain injury showed either occipital (13) or parieto-occipital (9) changes in white matter in both hemispheres on T2 scanning, typical of early-life hypoglycaemia.

**Table 1:** Clinical findings and MRI report from all patients considered to have possible missed hypoglycaemia.

	Clinical presentation	MRI findings
1.	Right-sided unprovoked seizures with secondary generalised seizures	Bilateral occipital gyral volume loss, more so on the right.
2.	Tip toe walking. Ex-preterm, severe intra-uterine growth restriction (IUGR)	There is abnormality in the left occipital lobe with what appear to be a focal area of gliosis and cortical atrophy, Periventricular high signal is noted in the occipital lobe on the right raising the possibility of localised gliosis.
3.	Developmental delay, dyspraxia	Bilateral symmetrical abnormal high intensity signal seen in the deep white matter of both occipital lobes with prominence in perivascular spaces.
4.	Increasing Head Circumference, Learning difficulties	T2/FLAIR high signal in the right occipital white matter extending from the periventricular deep white matter to the subcortical white matter, consistent with gliosis.
5.	Nystagmus, Learning difficulties	Gyral crowding involving occipital lobes worse on the left than the right with some prominence to the overlying CSF space.
6.	Developmental delay	Mild asymmetry of the ventricular system, with prominent right occipital horn and right occipital sulcal prominence compared to the left, presumably ex-vacuo dilatation of the right occipital horn due to ipsilateral occipital parenchymal volume loss.
7.	Learning difficulties	Bilateral cystic encephalomalacia/gliosis in the fronto-temporoparietal-occipital lobe.
8.	Developmental delay	Features suggestive of occipital lobe atrophy.
9.	Hypertonia in Lower Limb	Periventricular Leukomalacia with impaired myelination extending into the deep fronto-parietal white matter, prematurity related. Bilateral cortico-subcortical signal abnormality in the parieto-occipital lobes associated with volume loss.
10.	Microcephaly, Learning difficulties	Medial occipital lobe volume loss and high signal change within the white matter.
11	Weakness in right arm	Generalised prominence of extra-axial CSF over the left occipital lobe raising the possibility of some underlying atrophy.
12.	Microcephaly, drug resistant epilepsy	Mild prominence of cerebral and cerebellar sulci, more prominent in the parieto-occipital lobes bilaterally.
13.	Lethargy, floppy	Bilateral parieto-occipital volume loss + bilateral abnormality in the globi pallidi in association with mild cerebellar atrophy.
14.	Developmental delay, neonatal abstinence syndrome	Evidence of white matter loss and gyral crowding in both occipital lobes.
15.	Generalised seizure	High signal on the diffusion sequences in the parieto-occipital region.
16.	Recurrent apnoea	Bilateral symmetrical T2 high signal in the subcortical white matter in the parietal occipital lobes with diffusion changes and loss of cortical ribbon in same areas.
17.	Reduced GCS, meningitis	Bilateral occipital changes (Rt > Lt).
18.	Weakness and right-sided, severe delay in speech and language	Significant bilateral occipital lobe changes.
19.	Epilepsy, recent generalised tonic-clonic seizures	Bilateral medial occipital lobe subcortical white matter hyperintense change. Volume sequence shows some cortical volume loss on the right.
20	increasing sacral dimple	Encephalomalacia/gliosis with volume loss in the left parasagittal parieto-occipital lobes with cortical involvement.
21.	Hypoxic ischaemic encephalopathy, seizures	Bilateral slight increase T2 high signal in the parietal occipital white matter.
22	Poorly controlled epilepsy, learning difficulties	Bilateral parietal occipital abnormalities; also has left frontal lobe abnormality.

## Discussion

Our retrospective review of patients with radiological evidence of brain injury strongly suggests that a proportion sustained their brain damage due to unrecognised, early-life hypoglycaemia. Our findings support concerns of missed hypoglycaemia raised by patient organisations such as Congenital Hyperinsulinism International (6) and align with recent observations correlating MRI findings with a history of hypoglycaemia (8). Our findings also support hypoglycaemia as potential aetiology for unexplained neurodevelopmental abnormalities presenting in later life.

Our study provides initial but important evidence for a preventable cause of long-term neurodisability, adding support to calls for greater monitoring for the risk of hypoglycaemia in early life, particularly in the neonatal period. However, we accept that our study was retrospective, restricted to a single centre and that causal association, chiefly that with congenital hyperinsulinism, was not explored comprehensively. This study was further limited by incomplete information in a few cases, with potential for improved diagnostic pointers about the nature of hypoglycaemia. Nonetheless, absent information in a minority of patients does not digress from the importance of the main finding, that of missed and unrecognised hypoglycaemia leading to brain injury.

Our study was not focused on the comprehensive evaluation of the prevalence of hypoglycaemia related brain injury in early life or the association of the degree and duration of glycaemic excursion from normal for each child, as demonstrated in other studies (12). Instead, our study successfully focused on potential missed opportunities to detect early life hypoglycaemia.

Our study used typical scan findings based on occipital and parietal (or both) lobe changes to identify radiological evidence of hypoglycaemia. MR scan based radiological evidence is specific to early-life hypoglycaemia (12) with 82% positive predictive value in a large case series (9), suggesting that scan findings in our cohort did indeed reflect true evidence for hypoglycaemic brain injury. Other differentials of these MRI findings are possible but, given the high predictive value for hypoglycaemia, not worth visiting in depth. We did not use deep grey matter loss (for instance in the caudate nucleus) as another measure of hypoglycaemia (13) as our priority was specificity over sensitivity. While deep grey matter loss has been described, significant volume loss criteria have not been developed for use as a clinical tool. It is possible that future studies may identify grey matter parameters correlating with the extent and severity of neuroglycopaenia related brain injury.

While our study results provide insight into the difficulty of recognition of clinically important hypoglycaemia in early life, they do not provide information about safe levels of glucose in infancy, or those who should be considered for screening of hypoglycaemia. As our study design only incorporated the assessment of the initial scan report, disregarding later serial scan reports, radiological improvement was not documented. However, the absence of a longitudinal element in our study is not a fundamental flaw, as the successful cross-sectional determination of radiological evidence of hypoglycaemia was sufficient to address the study’s aim. Our study findings may be used as a steppingstone to design large multi-centric studies testing severity and duration of hypoglycaemia to ascertain the true prevalence of hypoglycaemia mediated brain injury. Nonetheless, the recognition of the existence of missed early-life hypoglycaemia, with potential for deleterious neurodevelopmental consequences, prompts the need for further development of neonatal hypoglycaemia screening practices. Additionally, our study findings point to the consideration for missed hypoglycaemia in diagnostic checklists for clinicians ordering MRI scans for neurodevelopmental abnormalities presenting in later life.

Although our study was not designed to address the true prevalence of missed opportunities for neonatal identification of hypoglycaemia, we have identified a significant number of hypoglycaemia related brain changes suggestive of early-life unrecognised hypoglycaemia. It is possible that our study missed other cases of hypoglycaemia; for this, a larger study involving multiple centres will be required. Such studies may require longitudinal follow-up to correlate the severity of neonatal hypoglycaemia and identify contributory aggravating factors to provide a fuller description informing causality. This study points to the need for greater awareness and the need to refocus on diagnostic frameworks for neonatal hypoglycaemia.

## Data availability statement

The original contributions presented in the study are included in the article/supplementary material. Further inquiries can be directed to the corresponding author.

## Author contributions

CW: Conceptualization, Formal analysis, Investigation, Methodology, Writing – original draft, Writing – review & editing. PG: Investigation, Writing – review & editing. KR: Investigation, Writing – review & editing. SW: Data curation, Writing – review & editing. MS: Writing – review & editing. AM: Data curation, Supervision, Writing – review & editing. IB: Methodology, Supervision, Writing – review & editing.
